# A novel saliva-based miRNA profile to diagnose and predict oral cancer

**DOI:** 10.1038/s41368-023-00273-w

**Published:** 2024-02-18

**Authors:** Jaikrishna Balakittnen, Chameera Ekanayake Weeramange, Daniel F. Wallace, Pascal H. G. Duijf, Alexandre S. Cristino, Gunter Hartel, Roberto A. Barrero, Touraj Taheri, Liz Kenny, Sarju Vasani, Martin Batstone, Omar Breik, Chamindie Punyadeera

**Affiliations:** 1 Saliva & Liquid Biopsy Translational Laboratory, Griffith Institute for Drug Discovery, Griffith University, Nathan, QLD Australia; 2 Department of Medical Laboratory Sciences, Faculty of Allied Health Sciences, University of Jaffna, Jaffna, Sri Lanka; 3 Menzies Health Institute, Griffith University, Gold Coast, QLD Australia; 4 School of Biomedical Sciences, Faculty of Health, Queensland University of Technology, Brisbane, QLD Australia; 5 Centre for Cancer Biology, Clinical and Health Sciences, University of South Australia & SA Pathology, Adelaide, SA Australia; 6 Institute of Clinical Medicine, Faculty of Medicine, University of Oslo, Oslo, Norway; 7 Department of Medical Genetics, Oslo University Hospital, Oslo, Norway; 8 Griffith Institute for Drug Discovery, Griffith University, Nathan, QLD Australia; 9 QIMR Berghofer Medical Research Institute, Statistics Unit, Brisbane, QLD Australia; 10 School of Public Health, The University of Queensland, Brisbane, QLD Australia; 11 School of Nursing, Queensland University of Technology, Brisbane, QLD Australia; 12 eResearch, Research Infrastructure, Academic Division, Queensland University of Technology, Brisbane, QLD Australia; 13 Department of Anatomical Pathology, Royal Brisbane and Women’s Hospital, Herston, QLD Australia; 14 Faculty of Medicine, The University of Queensland, Brisbane, QLD Australia; 15 Royal Brisbane and Women’s Hospital, Cancer Care Services, Herston, QLD Australia; 16 Department of Otolaryngology, Royal Brisbane and Women’s Hospital, Herston, QLD Australia; 17 Department of Oral and Maxillofacial Surgery, Royal Brisbane and Women’s Hospital, Herston, QLD Australia

**Keywords:** Oral cancer detection, Diagnostic markers, Predictive markers

## Abstract

Oral cancer (OC) is the most common form of head and neck cancer. Despite the high incidence and unfavourable patient outcomes, currently, there are no biomarkers for the early detection of OC. This study aims to discover, develop, and validate a novel saliva-based microRNA signature for early diagnosis and prediction of OC risk in oral potentially malignant disorders (OPMD). The Cancer Genome Atlas (TCGA) miRNA sequencing data and small RNA sequencing data of saliva samples were used to discover differentially expressed miRNAs. Identified miRNAs were validated in saliva samples of OC (*n* = 50), OPMD (*n* = 52), and controls (*n* = 60) using quantitative real-time PCR. Eight differentially expressed miRNAs (miR-7-5p, miR-10b-5p, miR-182-5p, miR-215-5p, miR-431-5p, miR-486-3p, miR-3614-5p, and miR-4707-3p) were identified in the discovery phase and were validated. The efficiency of our eight-miRNA signature to discriminate OC and controls was: area under curve (AUC): 0.954, sensitivity: 86%, specificity: 90%, positive predictive value (PPV): 87.8% and negative predictive value (NPV): 88.5% whereas between OC and OPMD was: AUC: 0.911, sensitivity: 90%, specificity: 82.7%, PPV: 74.2% and NPV: 89.6%. We have developed a risk probability score to predict the presence or risk of OC in OPMD patients. We established a salivary miRNA signature that can aid in diagnosing and predicting OC, revolutionising the management of patients with OPMD. Together, our results shed new light on the management of OC by salivary miRNAs to the clinical utility of using miRNAs derived from saliva samples.

## Introduction

Oral cancer is the sixteenth most common cancer worldwide, with a high prevalence in particular regions and certain ethnicities with predisposing lifestyles. The geographical clustering of its prevalence appears to be linked with high tobacco and alcohol usage and chewing of betel nut, often with lime, which is attributed as a major risk factor for OC. Oral squamous cell carcinoma (OSCC) accounts for over 90% of all OC cases. The global five-year survival rate of newly diagnosed OC patients is approximately 50%.^1^ Lack of community knowledge, diagnostic delays, and difficulties in seeking health assistance in some countries significantly lead to poor outcomes. Therefore, relevant, timely intervention may reduce the chance of loco-regional/distant metastasis and related complications.^2^ The early stages of OC are often asymptomatic as such current diagnostic strategies often fail to detect malignant lesions early.^2^ Treatment for OC is stage-dependent and often highly morbid. As such, identifying patients at their pre-cancerous stages provides an opportunity for timely treatment to sequester malignant transformation. More importantly, about 8% of OC develop from oral potentially malignant disorders (OPMD), a pre-cancer stage of OC.^3^ OPMDs can present as localised or widespread lesions affecting significant portions of the oral mucosa.^4^

Currently, histo-pathological assessment of biopsy samples is considered the gold standard for diagnosing OC and OPMD. However, it requires an invasive procedure, requiring trained surgical personnel, and the expertise of anatomical pathologists to ensure the most accurate diagnosis.^5^ An incisional biopsy only represents the exact area of the lesion being biopsied, and this limitation has encouraged scientists and clinicians to seek for alternative methods to account for tumour heterogeneity.^5^ In addition, less sensitive and specific methods such as vital staining, oral cytology, and optical imaging are in clinical practice, but each method has limitations.^6^ Despite the health impact of OC, there are no approved stand-alone biomarkers for early detection and prediction of OC. The development of a reliable non-invasive biomarker would play a decisive role in identifying OPMD patients who are likely to progress to invasive disease.

MicroRNAs (miRNAs) are small non-coding RNAs of approximately 19 to 22 nucleotides in length.^7,8^ They function as downstream regulators of gene expression at the transcriptional and post-transcriptional levels by mainly binding to sequence motifs located within the 3′ untranslated region (UTR) of mRNA. Usually, the extracellular miRNAs are released from cancer cells through vesicle trafficking and protein/ lipid carrier mechanisms into the body fluids.^9^ These miRNAs regulate cell proliferation, migration, invasion and angiogenesis.^9^ Studies have highlighted that miRNAs can function as either oncogenic or tumour suppressors in most cancers, including OC. Also, miRNA expression profiles are known to be tumour and tissue-specific.^10^ In addition, miRNAs are highly resistant to RNAase degradation and are more stable in body fluids.^11,12^

Recently, salivary diagnostics have received significant attention during the height of the COVID-19 pandemic.^13^ The utility of saliva as a potential diagnostic fluid offers a plethora of benefits, such as non-invasiveness, ease of accessibility, and convenience for multiple/ repeated collections.^14^ Regarding OC diagnosis, saliva is highly relevant and sensitive, as these tumours are in direct contact with saliva. Thus, tumour-associated cellular and biomolecular alterations can readily be detected in saliva.^15^

Recent developments in RNA sequencing and computational analysis have paved the way for transcriptome-wide biomarker discovery enabling the characterisation of tumours at their molecular level, including OC. Furthermore, the availability of high throughput, comprehensive, publicly available tumour tissue miRNA sequencing data in The Cancer Genome Atlas (TCGA) is beneficial in screening a diverse range of samples, thus, reducing bias in identifying potential biomarker targets. As such, this study aimed to combine next-generation sequencing data from saliva and TCGA datasets to identify miRNA signatures that can help differentiate between OC, OPMD and controls and validate them in an independent cohort.

## Results

### Clinicopathological features of participants

Our study design consisted of two phases: a discovery phase, and a validation phase. The clinicopathological characteristics of the participants who were recruited in the validation phase are summarised in Table 1. Additional details are available in Supplementary Table 1.

**Table 1:** Clinicopathological features of oral cancer, oral potentially malignant disorders and controls

Characteristics	Class	Oral cancer (*n* = 50), *n* (%)	OPMD (*n* = 52), *n* (%)	Controls (*n* = 60), *n* (%)
Demographical features				
Age/year	Mean [range]	64.8 [47–87]	63.4 [32–84]	67.4 [43–89]
Gender	Males	38 (76)	28 (53.8)	38 (63.3)
	Female	12 (24)	24 (46.2)	22 (36.7)
Risk factors				
Smoking status	Non-Smoker	7 (14)	18 (34.6)	26 (43.3)
	Ex-smoker	23 (46)	21 (40.4)	30 (50)
	Current smoker	8 (16)	11 (21.2)	4 (6.7)
Alcohol consumption	Non-drinker	3 (6)	23 (44.2)	25 (41.7)
	Ex-drinker	3 (6)	-	-
	Current drinker	22 (44)	27 (51.9)	35 (58.3)
Clinical details				
Location of cancer/site of lesion	Tongue	25 (50)	24 (46.2)	-
	Floor of Mouth	10 (20)	6 (11.5)	-
	Others	11 (6)	8 (15.3)	-
AJCC staging (8th edition)	Stage I	12 (27.9)	-	-
	Stage II	11 (25.6)	-	-
	Stage III	06 (14.0)	-	-
	Stage IVA	12 (27.9)	-	-
	Stage IVB	02 (4.7)	-	-
T stage	T1	14 (28)	-	-
	T2	17 (34)	-	-
	T3	3 (6)	-	-
	T4	9 (18)	-	-
N stage	N0	28 (56)	-	-
	N1	6 (12)	-	-
	N2	8 (16)	-	-
	N3	2 (4)	-	-
M stage	0	44 (88)	-	-
Treatment type	Surgery	25 (50)	-	-
	Surgery+Radiotherapy	15 (30)	-	-
	Surgery+Radiotherapy+Chemotherapy	1 (2)	-	-
	Chemotherapy	1 (2)	-	-
	Radiotherapy	1 (2)	-	-
Grade of dysplasia	Low grade	-	15 (28.8)	-
	High grade	-	12 (23.1)	-
	Lichenoid lesions	-	13 (25)	-

The clinicopathological features for some participants were unavailable: thus some of the numbers will not sum up to 100%

### Discovery phase

#### In-silico discovery phase—TCGA database

**Fig. 1:**
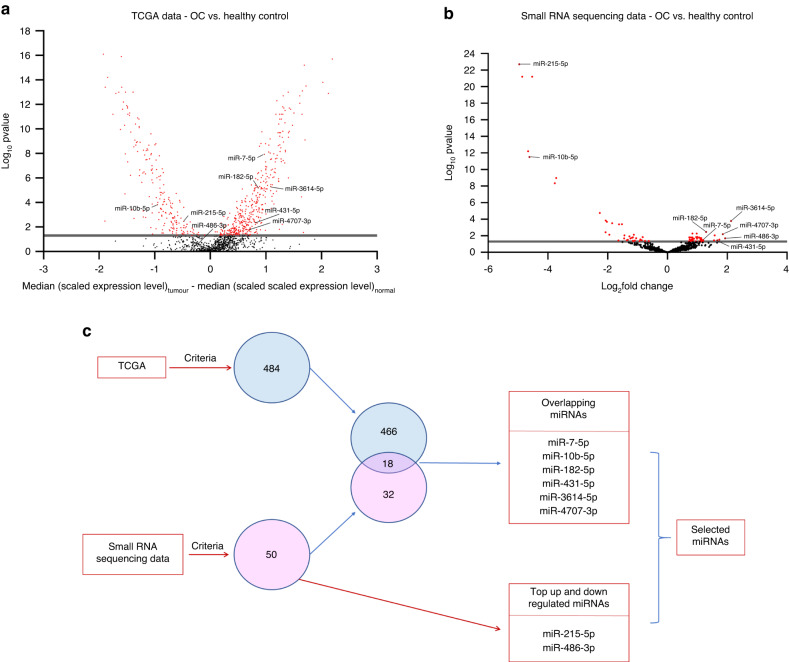
Differential expressions of eight microRNAs. **a**, **b** Volcano plots showing the differentially expressed miRNAs: **a** TCGA Data between HPV Negative OC (*n* = 160) and healthy controls (*n* = 30). **b** Small RNA sequencing data of saliva samples between HPV Negative OC (*n* = 12) and healthy controls (*n* = 6). Red dots indicate the significantly expressed miRNAs (Wilcoxon rank-sum test, *P* < 0.05). **c** Eight candidate miRNAs were selected

Candidate miRNAs were selected based on the differential expression levels between the normal healthy tissues and oral cavity tumour tissues (difference in median >0.5, <-0.5 and *P* < 0.05). We have identified 484 differentially expressed miRNAs between normal (*n* = 30) and OC tissue (*n* = 160) (Fig. 1a).

#### Small RNA sequencing of saliva samples

We assessed the small RNA from saliva samples from OC (*n* = 12), and controls (*n* = 6). The sequencing data showed that 50 miRNAs were differentially expressed between these two cohorts (log_2_fold-change >1.0, <-1.0 and *P* < 0.05) (Fig. 1b).

#### Identification of an 8-miRNA panel that discriminates oral cancer from controls

**Fig. 2:**
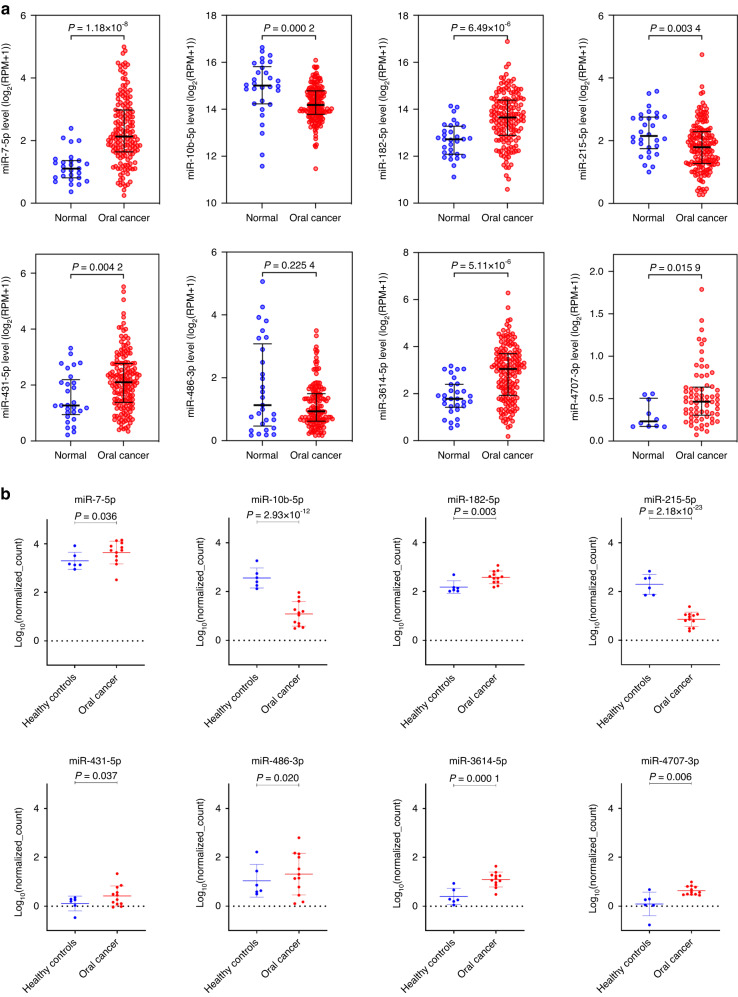
Differential expressions of selected miRNAs in the discovery phase. **a** Boxplots showing the differential expressions of selected miRNAs between OC (*n* = 160) and healthy controls (*n* = 30) in the TCGA dataset (Wilcoxon rank-sum test, *P* < 0.05). **b** Boxplots showing the differential expressions of the selected miRNAs between OC (*n* = 12) and healthy controls (*n* = 6) in our in-house small RNA sequencing data

A panel consisting of eight miRNAs was identified by comparing two datasets (TCGA and our in-house small RNA sequencing data). Among the eight miRNAs, six miRNAs, miR-7-5p, miR-10b-5p, miR-182-5p, miR-431-5p, miR-3614-5p and miR-4707-3p overlapped between both datasets and as such chose these miRNAs. In addition, we have also included the miRNA that was highly downregulated (miR-215-5p) and highly upregulated (miR-486-3p) miRNAs, from small RNA sequencing data of saliva samples (Fig. 1c) (Supplementary Table 2). The differential expressions of the selected miRNAs in the TCGA dataset and our in-house small RNA sequencing data have been illustrated in Fig. 2a, b, respectively. Using the above-mentioned eight miRNAs, we have developed a panel that was validated in the next phase.

### Selection of a suitable normaliser for miRNA quantitative reverse transcription PCR (RT-qPCR)

Five reference miRNAs (miR-16-5p, miR-191-5p, miR-484, SNORD 96A, and U6 snRNA) were selected based on the literature.^16–18^ The stability of these miRNAs was validated in saliva samples from controls (*n* = 60), OPMD (*n* = 52) and oral cancer (*n* = 50) using RT-qPCR. Among the putative reference miRNAs, miR-191-5p was the most stable miRNA based on RT-qPCR data. However, according to (Minimum Information for Publication of Quantitative Real-Time PCR Experiments) MIQE guidelines,^19^ the most stable combination of reference miRNAs was identified using NormFinder, which includes miR-191-5p, miR-484 and SNORD 96A (Table 2). The arithmetic mean of these three miRNAs was used as the normaliser for saliva samples and tissue samples.

**Table 2:** Results of NormFinder for the selection of reference gene/s for normalisation of RT-qPCR results

Gene name	Stability value		
miR-16-5p	0.023		
miR-191-5p	0.012		
miR-484	0.013		
SNORD96A	0.016		
U6 snRNA	0.024		
Average (miR-16-5p, miR-191-5p, miR-484)	0.007		
Average (miR-16-5p, miR-191-5p, SNORD96A)	0.014		
Average (miR-16-5p, miR-191-5p, U6 snRNA)	0.004		
Average (SNORD96A, miR-191-5p, U6 snRNA)	0.006		
***Average (miR-191-5p,miR-484, SNORD96A)***	***0.004***		
Average (miR-191-5p,miR-484, U6 snRNA)	0.011		
Average (miR-484, SNORD96A,U6 snRNA)	0.011		
**Intragroup variation**			
**Group identifier**	**OPMD**	**Controls**	**Oral cancer**
miR-16-5p	0.004	0.003	0.002
miR-191-5p	0.001	0.001	0.001
miR-484	0.001	0.001	0.001
SNORD96A	0.003	0.002	0.003
U6 snRNA	0.004	0.004	0.005
Average (miR-16-5p, miR-191-5p, miR-484)	0.001	0.000	0.000
Average (miR-16-5p, miR-191-5p, SNORD96A)	0.001	0.000	0.001
Average (miR-16-5p, miR-191-5p, U6 snRNA)	0.000	0.000	0.000
Average (SNORD96A, miR-191-5p, U6 snRNA)	0.000	0.000	0.000
***Average (miR-191-5p,miR-484, SNORD96A)***	***0.000***	***0.000***	***0.000***
Average (miR-191-5p,miR-484, U6 snRNA)	0.000	0.000	0.000
Average (miR-484, SNORD96A,U6 snRNA)	0.000	0.000	0.001
**Intergroup variation**			
**Group identifier**	**OPMD**	**Controls**	**Oral cancer**
miR-16-5p	0.022	0.004	-0.026
miR-191-5p	0.009	-0.001	-0.007
miR-484	-0.011	-0.001	0.013
SNORD96A	0.013	0.000	-0.013
U6 snRNA	-0.027	0.001	0.027
Average (miR-16-5p, miR-191-5p, miR-484)	0.005	0.000	-0.005
Average (miR-16-5p, miR-191-5p, SNORD96A)	0.014	0.001	-0.015
Average (miR-16-5p, miR-191-5p, U6 snRNA)	-0.002	0.001	0.001
Average (SNORD96A, miR-191-5p, U6 snRNA)	-0.004	0.000	0.004
***Average (miR-191-5p,miR-484, SNORD96A)***	***0.003***	***-0.001***	***-0.002***
Average (miR-191-5p,miR-484, U6 snRNA)	-0.012	-0.001	0.012
Average (miR-484, SNORD96A,U6 snRNA)	-0.010	-0.001	0.010
**Best gene**	Average (miR-191-5p,miR-484, SNORD96A)		
**Stability value**	0.004		

The most stable reference gene/combination has the least stability value which is calculated based on the inter-group and intra-group variations

Italicized values: the Normfinder software does not report the level of significance for the stability values. So we are unable to report the same

### Validation phase

#### Differential expression levels of eight miRNAs in saliva samples

**Fig. 3:**
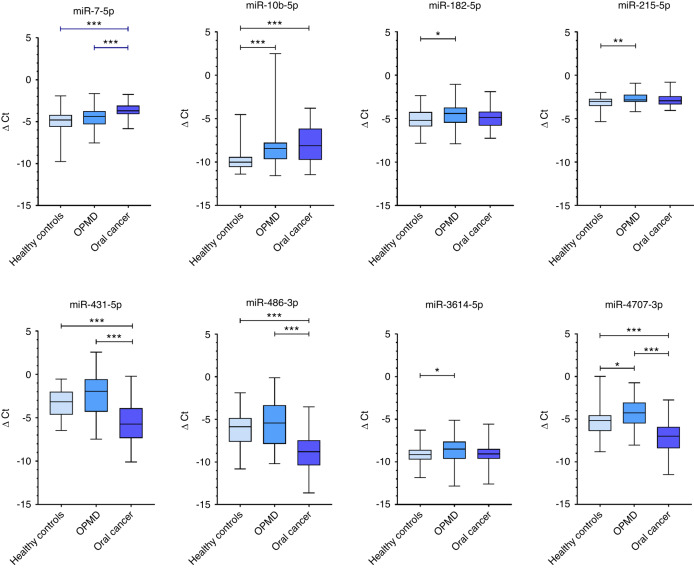
Boxplots of eight-miRNA expressions in saliva samples of OC, OPMD, and controls using RT-qPCR. The differential expressions of miRNAs were shown (one-way ANOVA, Tukey’s HSD, ***P ≤ 0.001, **P ≤ 0.01, *P ≤ 0.05)

The differential expression levels of the eight miRNAs in saliva samples from OC (*n* = 50), OPMD (*n* = 52) and controls (*n* = 60) (Fig. 3).

#### Oral cancer diagnosis using a panel of eight miRNAs

**Fig. 4:**
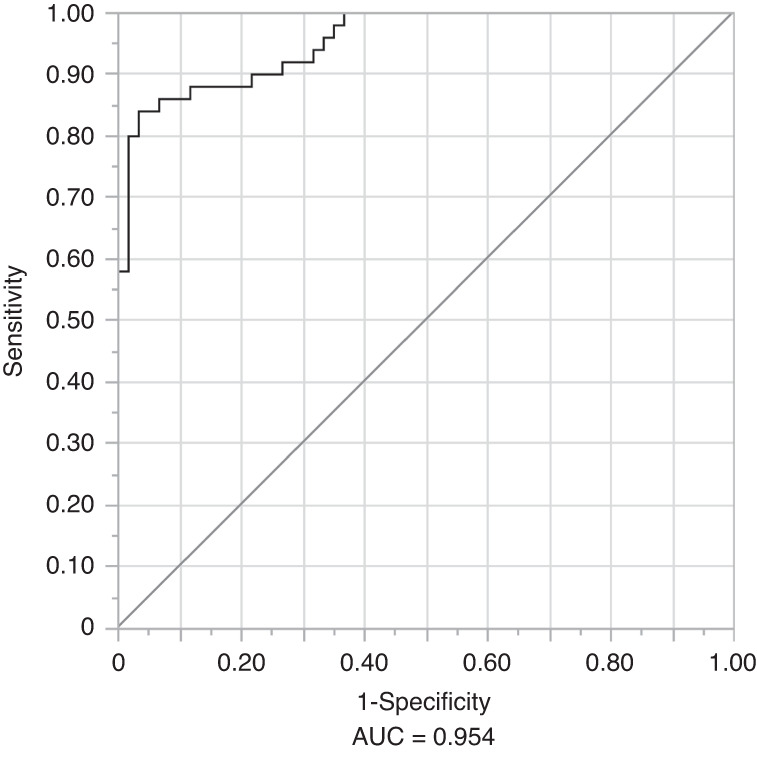
The diagnostic performance of eight-miRNA signature in the validation phase (OC vs. controls). Receiver operating characteristic curves for detecting oral cancer

The diagnostic potential of the eight-miRNA panel was validated in a cohort of 50 OC and 60 controls using RT-qPCR. Among these miRNAs, expression levels of miR-7-5p, miR-10b-5p, miR-431-5p, miR-486-3p, and miR-4707-3p showed statistically significant differences between cohorts. miR-7-5p and miR-10b-5p were upregulated, while miR-431-5p, miR-486-3p, and miR-4707-3p were downregulated in saliva samples from OC patients compared to controls (*P* < 0.05, ANOVA, Tukey’s HSD test). A Least Absolute Shrinkage and Selection Operator (LASSO) logistic regression model of OC vs. healthy controls was fit for these eight miRNAs. The model minimising the AICc criterion included all eight miRNAs (Table 3). These eight miRNAs were able to detect OC with an Area Under Curve (AUC) of 0.954, a sensitivity of 86%, with specificity fixed at 90%, a positive predictive value (PPV) of 87.8%, and a negative predictive value (NPV) of 88.5% (probability threshold = 0.537) (Fig. 4).

**Table 3:** Odds ratios for a panel of eight salivary miRNAs that can diagnose oral cancer

miRNA	Odds ratio	95% CI (confidence interval)		*P*-value
miR-10b-5p	3.91	2.02	7.57	<0.000 1
miR-3614-5p	6.09	1.76	21.04	0.004 3
miR_431-5p	0.40	0.21	0.76	0.005 0
miR-486-3p	0.69	0.47	1.02	0.064 0
miR-182-5p	1.70	0.69	4.20	0.249 1
miR-215-5p	2.20	0.56	8.66	0.261 5
miR-7-5p	1.64	0.69	3.90	0.264 0
miR-4707-3p	0.79	0.39	1.57	0.493 6

LASSO logistic regression model was performed to calculate the odds ratios. Confidence intervals were determined at a 95% level

**Fig. 5:**
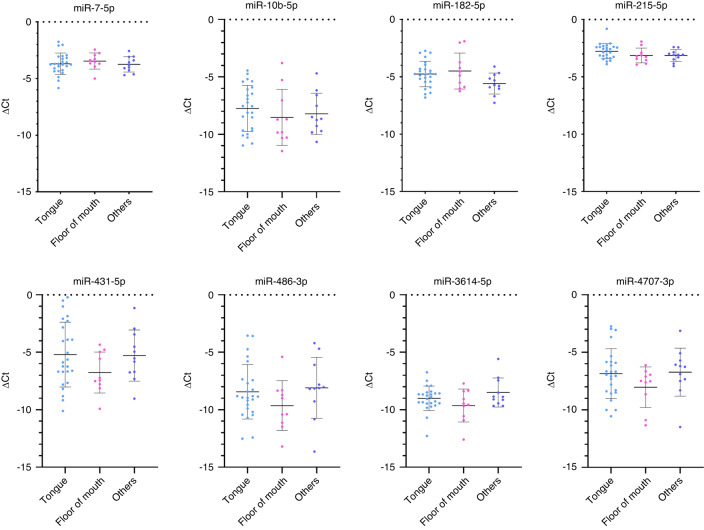
Differential expressions of salivary miRNAs based on the location of the tumour. Tongue, *n* = 25; Floor of mouth, *n* = 10; others, *n* = 11 (others include buccal mucosa, retromolar trigone, hard palate, maxillary alveolus)

Additionally, we analysed the differential expressions of the eight salivary miRNAs based on the tumour location. However, our analysis did not reveal any statistically significant difference in these miRNAs among the tumour locations (ANOVA, *P* > 0.05) (Fig. 5).

#### A saliva-based four-miRNA panel can discriminate patients with oral cancer from oral potentially malignant disorder patients

**Fig. 6:**
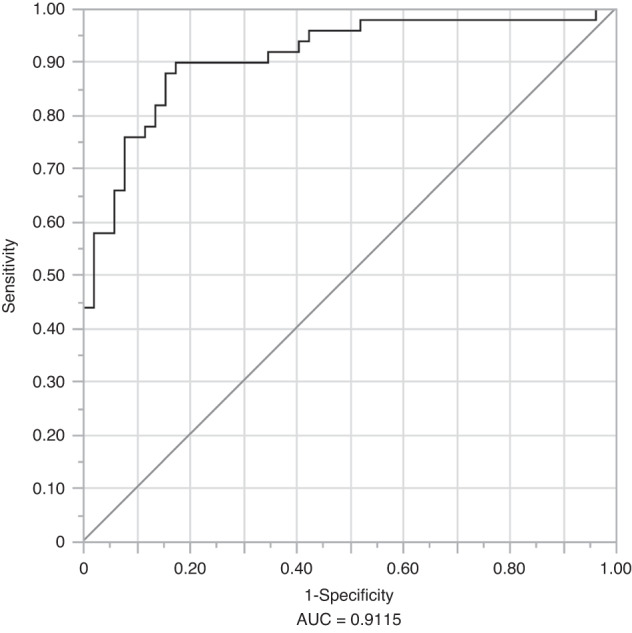
The area under curve (AUC) of the four-miRNA panel for discrimination between OC and OPMD. The AUC value is calculated using the receiver operating characteristic analysis

The OPMD cohort includes patients with low-grade dysplasia, high-grade dysplasia and lichenoid lesions. We found miR-7-5p, miR-431-5p, miR-486-3p and miR-4707-3p to be differentially expressed in saliva samples from OC (*n* = 50) and OPMD (*n* = 52) patients. Except for miR-7-5p, the other three miRNAs were upregulated in OPMD patients when compared to OC patients. A LASSO logistic regression model of OC vs. OPMD was fit for these eight miRNAs. The model minimising the AICc criterion included four miRNAs (miR-7-5p, miR-10b-5p, miR-215-5p, and miR-4707-3p) (Table 4). This panel achieved an AUC of 0.9115, with sensitivity fixed at 90%, specificity was 82.7%, PPV of 74.2% and NPV of 89.6% (probability threshold = 0.450) (Fig. 6).

**Table 4:** Odds Ratios for four-miRNA panel: oral cancer vs. oral potentially malignant disorders

Source	Odds Ratio	95% CI		*P*-value
miR-4707-3p	0.41	0.26	0.64	<0.000 1
miR-7-5p	2.00	1.08	3.70	0.026 8
miR-215-5p	0.34	0.13	0.90	0.029 9
miR-10b-5p	1.42	0.99	2.04	0.057 5

The odds ratio presented in the table represents the ability of the four miRNAs to discriminate between OC and controls. The LASSO logistic regression model was performed to calculate the odds ratios. Confidence intervals were determined at a 95% level, statistical significance was considered at *P* < 0.05

#### A panel of four saliva-based miRNA can effectively diagnose patients with oral potentially malignant disorders

**Fig. 7:**
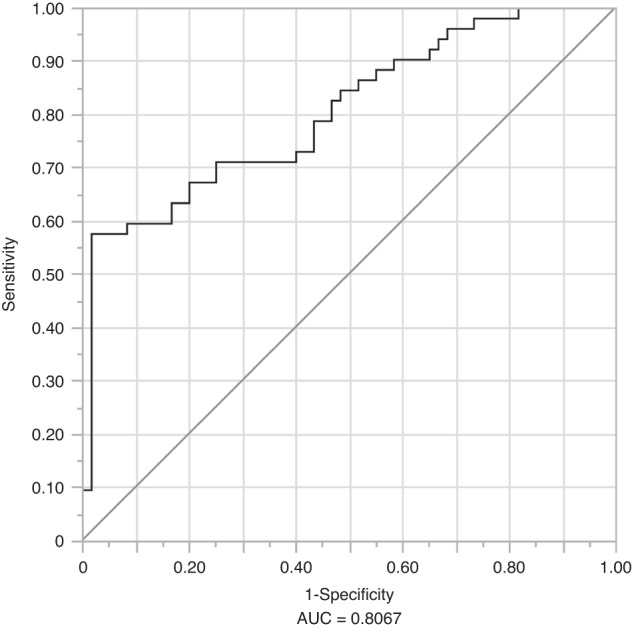
The discriminative performance of four-miRNA signature in the validation phase. Receiver operating characteristic curves and its fitted probability model for distinguishing OPMD patients from controls

We found that miR-10b-5p, miR-182-5p, miR-215-5p, miR-3614-5p, and miR-4707-3p to be differentially expressed (*P* < 0.05, ANOVA) between saliva samples from OPMD (*n* = 52) and controls (*n* = 60). A LASSO logistic regression model was used to distinguish between OC and OPMD. Upon minimising the AICc criterion, the model included four miRNAs as the most significant markers (miR-10b-5p, miR-182-5p, miR-215-5p, and miR-3614-5p) (Table 5). We were able to discriminate OPMD from control using the saliva-based four-miRNA with an AUC of 0.807, a sensitivity of 59.6%, with specificity fixed at 90%, PPV of 83.8%, and NPV of 72% (probability threshold = 0.576) (Fig. 7).

**Table 5:** Odds Ratios for four-miRNA panel: oral potentially malignant disorders vs. controls

Source	Odds ratio	95% CI		*P*-value
miR_3614_5p	1.78	1.21	2.61	0.003 5
miR_10b_5p	1.83	1.19	2.81	0.005 9
miR_215_5p	2.73	1.19	6.26	0.018 0
miR_182_5p	1.51	1.02	2.24	0.041 9

The LASSO logistic regression model was performed to calculate the odds ratios. Confidence intervals were determined at a 95% level, statistical significance was considered at *P* < 0.05

#### The expression levels of candidate miRNAs in tissue samples

**Fig. 8:**
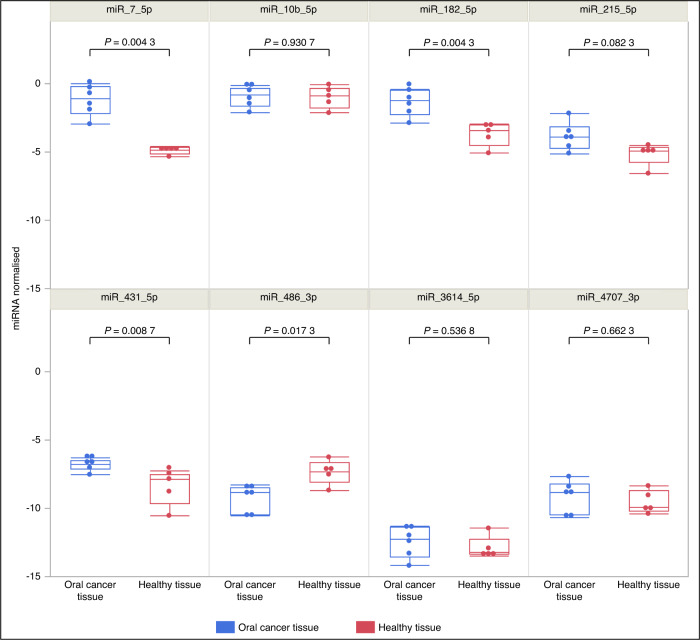
Boxplots illustrating the expression levels of miRNAs in tissue samples. It is evident that miR-7-5p, miR-182-5p, miR-431-5p and miR-486-3p exhibit significant expression changes between tumour tissue (*n* = 6) and healthy tissue (*n* = 5)

To confirm the association between candidate miRNAs and OC we then investigated the expression levels of them in OC tissues (*n* = 6) and healthy oral tissue samples (*n* = 5). The results demonstrated that miR-7-5p, miR-182-5p, and miR-431-5p were significantly upregulated, whereas miR-486-3p was significantly downregulated in OC tissues compared to healthy oral tissue samples (Wilcoxon rank-sum test, *P* < 0.05) (Fig. 8). Moreover, miR-7-5p and miR-486-3p exhibited a similar expression pattern as observed in saliva samples.

#### Localisation of miR-7-5p in FFPE tissue samples

**Fig. 9:**
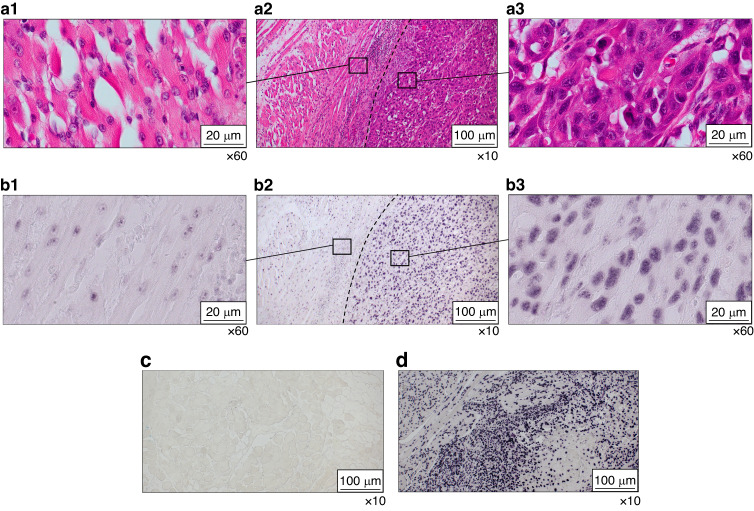
Localisation of miR-7-5p in FFPE tissue samples using miRNA in situ hybridisation. **a1** H&E-stained Adjacent Normal Region of the test sample (x60), **a2** Overview of H&E-stained section of the test sample (x10), **a3** H&E-stained tumour region of test sample (x60). **b1** miRNA in situ hybridisation of Adjacent Normal Region of the test sample (x60), **b2** Overview of miRNA in situ hybridisation of test sample (x10), **b3** miRNA in situ hybridisation of Tumour Region of Test sample (x60). It is evident that the tumour region exhibits stronger staining compared to the adjacent normal region, demonstrating the overexpression of miR-7-5p in the tumour region. **c** Negative control stained with DIG-labelled scrambled miRNA probe (x10). **d** Positive control using DIG-labelled U6 snRNA probe (x10)

Based on the salivary and tissue expression levels, we selected the most differentially expressed miRNA, miR-7-5p, to determine its expression patterns in tumour tissue. miRNA in situ hybridisation demonstrated that miR-7-5p is overexpressed in the tumour area compared to the adjacent normal region, confirming that overexpression of miR-7-5p is associated with tumour expression patterns (Fig. 9a, b, c, d and Supplementary Fig. 1).

#### Salivary miR-7-5p can diagnose oral cancer and differentiate oral cancer from oral potentially malignant disorders

**Fig. 10:**
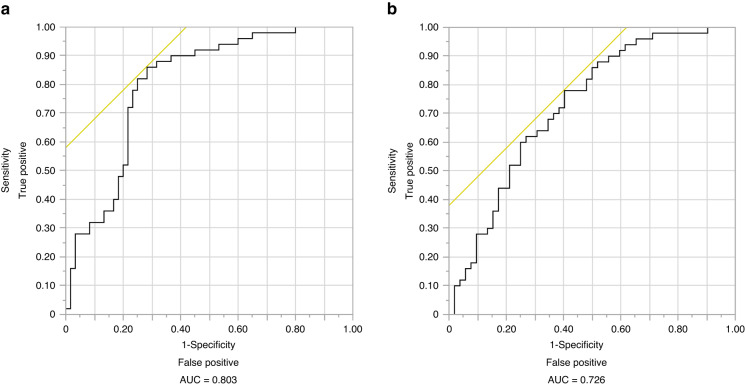
The discriminative performance of salivary miR-7-5p in the validation phase. **a** Oral cancer vs. Healthy controls, **b** oral cancer vs. oral potentially malignant disorders. The AUC value is calculated using receiver operating characteristic analysis

In light of these exciting findings of differential expressions of salivary and tissue miR-7-5p, we found that salivary miR-7-5p can be used as a potential independent marker for OC diagnosis and to differentiate OC from OPMD. The efficiency of miR-7-5p for diagnosing OC (vs. healthy controls) based on AUC, sensitivity, specificity, PPV, and NPV was 0.803, 70%, 78%, 73%, and 76%, respectively (odds ratio = 3.55) (Fig. 10a). In addition, miR-7-5p distinguishes OC from OPMD patients with an AUC of 0.726, a sensitivity of 96%, a specificity of 35%, a PPV of 59%, and an NPV of 90% (Odds ratio = 207.96) (Fig. 10b). However, the expression levels were not significant between OPMD and healthy controls.

#### Salivary miRNA panel can predict the presence or risk of oral cancer in patients with high-grade oral potentially malignant disorders

**Fig. 11:**
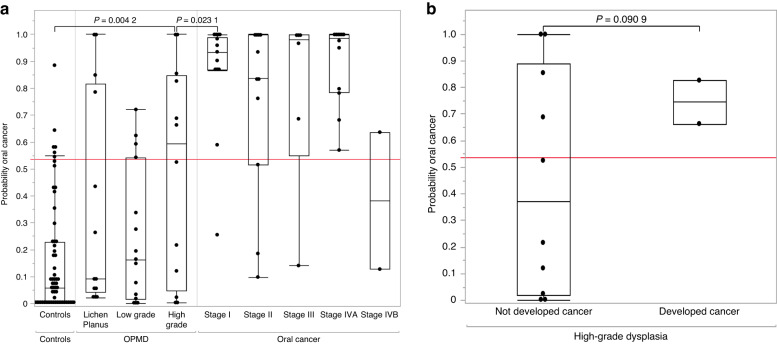
**a** Boxplots of risk probability scores were generated for healthy controls, patients with oral potentially malignant disorders (lichen planus, low grade and high-grade dysplasia) and oral cancer (stage I to IV). The saliva-based miRNA risk probability score effectively discriminates high-grade dysplasia from both Stage I oral cancer and controls. The risk score follows a gradual increase from mild dysplasia to the malignant stages except in Stage II and Stage IVB (Tukey’s HSD test, *P* ≤ 0.05). **b** Boxplots highlighting a higher risk probability score in patients with high-grade dysplasia who have developed oral cancer. It is evident that patients who have developed oral cancer have a relatively high-risk probability score compared to those who have not

The OPMD cohort was subcategorised into low grade and high grade based on the severity of dysplasia, while the OC cohort was subcategorised according to their American Joint Committee on Cancer (AJCC) staging (8th edition). Based on the eight-miRNA diagnostic model (OC vs. controls), we calculated the probability score for each participant. Patients with high-grade dysplasia had significantly higher probability scores than controls (*n* = 12, *P* = 0.004, Tukey’s HSD). Moreover, there was a significant difference (*P* < 0.05) in the risk score between Stage I OC patients and those with high-grade dysplasia. However, the scores were not significantly different between patients with lichenoid lesions or low-grade dysplasia and controls (Fig. 11a). During the study period, two patients initially diagnosed as high-grade dysplasia on incisional biopsy were found to have superficially invasive squamous cell carcinoma (SCC) on excision within 6 weeks of initial biopsy. According to our developed algorithm, their baseline risk probability scores were 0.66 and 0.82 (cut-off for OC diagnosis = 0.537), which were relatively higher than other patients with high-grade dysplasia with no evidence of invasive SCC on excision (Fig. 11b). Therefore, this miRNA signature could detect SCC in patients where biopsy underestimated the extent of disease.

## Discussion

OC is a heterogeneous multifactorial disease resulting from genetic and epigenetic alterations. OC is challenging to manage due to its aggressive nature, high metastatic rate, and late diagnosis leading to poor 5-year overall survival rates. In addition, a considerable proportion of patients (3% – 50%) with OPMD are at high risk of transforming into invasive carcinoma.^20^ In particular, patients with high-grade dysplasia (around 12%) are at risk for malignant transformation.^21^ Early detection and timely targeted therapy are well-known strategies for improving patient outcomes. As such, recent research has focused on identifying biomarkers for the diagnosis and prognosis of OC using a liquid biopsy approach. An ideal biomarker should have a high sensitivity and specificity through non-invasive, simple, and cost-effective methods, such as human saliva.

Our unique approach of combining TCGA tumour tissue miRNA sequencing data and salivary small RNA sequencing data to identify miRNAs led to the development of a robust miRNA-based panel. Furthermore, choosing the differentially expressed miRNAs in TCGA data that overlapped with saliva sequencing data and the inclusion of most up and downregulated miRNAs further increased the efficiency of our miRNA panel. More importantly, including overexpressed and under-expressed miRNAs further strengthened our biomarker discovery approach. In contrast, most previously published studies have considered only upregulated miRNAs.^17^ Furthermore, our systematic approach of selecting five miRNA reference genes to normalise RT-qPCR data ensures the reliability of the miRNA expression analysis. As a result, a combination of three highly stable miRNAs was considered as reference genes for normalising miRNA RT-qPCR quantification. Also, using LNA-based technology for the RT-qPCR further ensures the reliability of the expressions of miRNAs.

Regarding the non-invasive diagnosis of OC, the 8-miRNA signature achieved a diagnostic efficiency with an AUC, sensitivity, and specificity of 0.954, 86%, and 90%, respectively. Further validation of our candidate miRNAs in tissue samples confirmed that most of our candidate miRNAs exhibited similar expression patterns as those observed in saliva. However, due to the limited sample size, a few miRNAs did not show significant and similar patterns as those of saliva. For instance, miR-7-5p and miR-486-3p demonstrated similar patterns observed in saliva, indicating their association with the tumour. Moreover, the localisation of miR-7-5p in FFPE tissue samples further confirms its association with the tumour.

Furthermore, the most clinically relevant finding was the development of a risk probability score to detect and stratify patients at high risk of developing OC. Notably, patients with high-grade dysplasia are more prone to malignant transformation. Several studies have demonstrated that early detection and timely targeted therapy could be the best strategy to improve patient outcomes. Concurrently, our risk probability score predicted the presence of OC in two patients with OPMD on biopsy. In this context, two potential scenarios are conceivable. Firstly, the patients might have had malignant transformation (tumour) from the beginning and the first biopsy might not have represented the entire area of the tumour tissue, a phenomenon attributed to sampling error. This is one of the downsides of the current diagnostic methods. Secondly, as per the biopsy results, the patient might have transformed into OC in four to six weeks, which is less likely. However, we considered biopsy results as the gold standard and interpreted them accordingly. In any of the above scenarios, the risk probability score can be used to predict the OC risk in OPMD patients and if the score is on the higher side, it may lead the clinician to suspect a sampling error and proceed to more definitive treatment rather than surveillance. This finding could be a game changer in the management of high-risk OPMD patients, as this test could be used as a screening test to predict the presence or risk of OC in high-risk OPMD patients. However, we acknowledge that there are four patients with risk probability scores higher than the cut-off who have not yet developed OC. This may be attributed to the fact that we only collected patient outcomes during the study period. Nevertheless, we are continuously monitoring these patients to track their progress. Also, we acknowledge that the sample size of OPMD patients who had no evidence of OC in the first biopsy and subsequently diagnosed with OC in the second biopsy (*n* = 2) is insufficient to credit the findings completely. However, the findings of this study can serve as the foundation to pave the way for future studies including larger sample size. Another clinically relevant finding was the development of a four-miRNA signature to discriminate OPMD patients from OC. The discriminative efficiency of the four-miRNA signature based on AUC, sensitivity, and specificity was 0.9115, 90%, and 82.7%, respectively. miR-4707-3p demonstrated the highest discriminative efficiency, followed by miR-7-5p, miR-215-5p and miR-10b-5p.

Similar studies have reported the utilisation of salivary miRNAs for the diagnosis of OC. Koopaie et al. reported that both miR-15a and miR-16-1 were downregulated in saliva samples of OSCC patients (*n* = 15) compared to healthy controls (*n* = 15). miR-15a showed a sensitivity and specificity of 93.3% and 86.67%, respectively. In contrast, miR-16-1 showed a sensitivity and specificity of 86.67% and 92.33%, respectively. miR-15a shows more sensitivity than our miRNA signature in discriminating OSCC from controls, which may be due to the low sample number recruited in their study.^22^ Similarly, Romani et al. reported that a panel of miR-106-5p, miR-423-5p, and miR-193b was able to distinguish OC (*n* = 55) from healthy controls (*n* = 39) with an AUC of 0.98, sensitivity of 97.4% and specificity of 94.2%. Their study considered only the upregulated miRNAs, whereas we included both upregulated and downregulated ones to eliminate bias in selecting biomarkers. Duz et al. reported salivary miR-139-5p as a biomarker for the early detection of tongue squamous cell carcinoma.^23^ However, the discriminative efficiency of their study is lower than our study. Furthermore, Yap et al. reported a risk score combining miR-21-5p, miR-100-5p, and let-7-5p that could be used to assess the risk of OSCC.^24^ However, their AUC (0.868) and specificity (81.5%) were less than in our present study.

Overall, the results of the present study were consistent with other recent studies indicating the possibilities of using miRNAs as diagnostic and predictive biomarkers. However, except for some miRNAs reported in the present study, others have not been previously reported in OC, and we experimentally highlight their discriminative potential for the first time. For instance, Chou et al. found that miR-486-3p acted as a tumour-suppressive miRNA in OC by targeting the well-known oncogene DDR1.^25^ They reported that miR-486-3p is downregulated in tumours compared to their matched normal adjacent tissues. Our results also show a downregulation in saliva samples which is in concordance with the previous results. Similarly, Li et al. reported that miR-182-5p promoted the growth of OC by targeting CAMK2N1, thus functioning as an oncogenic miRNA.^26^ Even though we could not find a significant difference in the levels of miR-182-5p in saliva samples between OC and controls in this study, there is a notable upregulation in the OC cohort. Since miRNAs are secreted from cancer cells into saliva, when miRNAs are downregulated in the tumour, their secretion into saliva can also be reduced, resulting in the downregulation in saliva and vice versa. In contrast, miR-431-5p was reported to be downregulated in OC tissue when compared to adjacent normal tissue and act as a tumour suppressor in tongue squamous cell carcinoma.^27^ However, our results demonstrate that miR-431-5p is upregulated in OC tissues and downregulated in saliva samples of OC patients. This discrepancy can be attributed to several confounding factors, such as subtypes of OC, variations in tumour microenvironment, and stage of cancer. Notably, the previous study has exclusively included patients with tongue squamous cell carcinoma patients. However, our study included tumours from various anatomical sites of the oral cavity (floor of the mouth, buccal mucosa, maxillary alveolus, retromolar trigone, and hard palate). In addition, the expression levels of miR-431-5p can exhibit stage-dependent variations in OC. Unfortunately, we could not find the cancer stage of the previous study’s participants.

Additionally, variations in the composition of the tumour microenvironment should be considered, which can impact the expression of miRNAs.^28^ Accordingly, disparities in the tumour microenvironment between the samples of our study and the previous study could alter the expression patterns of miR-431-5p. Notably, our findings corroborate with the TCGA tumour tissue data, which indicated an upregulation of miR-431-5p in OC. The concordance with a relatively larger dataset provides additional validation of our findings. Other miRNAs from our panel have not been reported previously in oral cancer, but some have been reported as potential biomarkers or regulators in other cancer types. miR-7-5p was reported as a tumour suppressor in non-small cell lung cancer, hepatocellular carcinoma, and pancreatic ductal adenocarcinoma and was involved in enhancing temozolomide sensitivity in drug-resistant glioblastoma cells.^29–32^ Similarly, miR-215-5p was reported as a tumour-suppressor multiple myeloma and as a biomarker of diagnostic importance in osteosarcoma.^33–35^ Concurrently, miR-3614-5p was reported as an oncogene in hepatocellular carcinoma, and non-small cell lung cancer. In contrast, it was identified as a tumour-suppressor in cadmium-induced breast cancer^36–38^ whereas miR-4707-3p was reported in oesophageal carcinoma.^39^ miR-10b-5p was reported as a regulator of PIEZO1 in breast cancer and as a tumour-suppressor in primary hepatic carcinoma.^40,41^

Although our miRNA signatures were robust in diagnosing and predicting OC, we acknowledge the discrepancies in the expression levels between small RNA sequencing data and the validation phase using RT-qPCR. This may be due to the small cohort of patients used in the discovery phase. Furthermore, we could not trace the clinicopathological details of some patients as they were unavailable in the healthcare provider’s record, and we collected outcomes data for OPMD patients only during the study period.

To conclude, we have discovered and validated a non-invasive salivary miRNA panel to early diagnose OC. Furthermore, we have developed a risk probability score to stratify the patients with high-grade dysplasia and Stage I OC, thus, for the first time developing an algorithm to predict the presence or risk of OC in OPMD patients. Nevertheless, further validation of the reported salivary miRNA signatures in multi-centred clinical trials is warranted prior to clinical uptake.

## Materials and methods

### Study design and research ethics

This is a two-phase study that involves biomarker discovery and a validation phase. We analysed 18 saliva (next-generation sequencing) and 190 tumour tissue (TCGA dataset) data in the discovery phase. Salivary and tumour/ healthy oral tissue miRNA expression levels were analysed in OC, OPMD patients, and controls in the validation phase. Treatment naïve OC and OPMD patients were recruited from HNC clinics at the Royal Brisbane and Women’s Hospital (RBWH). Clinical research coordinators approached the eligible patients, and informed consent was sought. Controls were recruited from the general population. Ethical clearance was obtained from the Metro South Human Research Ethics Committee (HREC Reference number: HREC/12/QPAH/381).

### Sample collection, transportation and temporary storage

Saliva samples were collected and stored as previously reported.^18^ The demographic and other details regarding the risk factors of the patients/controls were obtained through a brief questionnaire. Tissue samples were collected in Qiazol and stored at -80°C.

### Small RNA extraction

The NucleoSpin miRNA isolation kit (Macherey-Nagel) was used to isolate miRNA from saliva and tissue samples. Extraction of miRNAs was performed as previously reported, 300 µL of saliva sample was used for isolation.^18,42^ Tissue samples were homogenised using a sterilised mortar and pestle. Samples were crushed into a fine powder and mixed with 1.5 mL of Qiazol (Qiagen) and extraction of miRNAs was followed as for saliva samples.

### Biomarker discovery phase

#### MicroRNA differential expression analyses using The Cancer Genome Atlas (TCGA) data

TCGA miRNA expression and clinical data from the head and neck cancer (HNC) dataset were downloaded from the Genomic Data Commons Data Portal (https://portal.gdc.cancer.gov). miRNA data comprised mature-strand expression levels, which were log_2_-transformed according to the formula below.

$$m={\log }_{2}\left(\left(\mathop{\sum }\limits_{i=1}^{i=n}{RPM}\right)+1\right)$$

Herein, m is the mature-strand miRNA expression level, which comprises the sum of the levels of all isoforms, if there are multiple; i represents the isoform transcripts number; n denotes the total number of isoform transcripts; and RPM represents the number of isoform transcript reads per million. For each normal and tumour tissue sample, its anatomical site was identified from matching clinical data from TCGA. Samples were identified as human papillomavirus positive (HPV+), negative (HPV-) or unknown using a combination of information in TCGA clinical data and previously reported HPV status.^43^ The analysis included the comparison of miRNA expression changes across normal tissues (*n* = 30) compared to HPV- tumour samples (*n* = 160). For differential expression analyses, within each defined group, miRNA expression levels were scaled, i.e., normalised on a scale from 0 to 1, and for each miRNA, a Wilcoxon rank-sum test was applied to determine whether the scaled expression levels were statistically significantly different.^44^ Volcano plots were generated by plotting the differences between the medians of the scaled expression levels to the –log_10_-transformed *P* values.

#### Small RNA sequencing of saliva samples

Small RNA sequencing was carried out in saliva samples collected from OC (*n* = 12), and controls (*n* = 6). Next-generation sequencing was carried out at BGI Genomics (New Territories, Hong Kong). Sequencing was performed using technologies such as combinatorial probe-anchor synthesis (cPAS), linear isothermal rolling-circle replication, and DNA nanoballs (DNB™). Unique molecular identifiers (UMIs) were used for accurate quantification.^45^

Raw small RNA-seq NGS data was quality assessed and trimmed using Trim-Galore (https://github.com/FelixKrueger/TrimGalore) retaining bases with *Q* > 20. High-quality trimmed reads were mapped onto both miRNA mature and complementary star sequences (miRBase release 22.1)^46^ using Bowtie 1.1.2^47^ allowing up to one mismatch. Mapping statistics were extracted using SAMtools.^48^ miRNA counts for all samples were merged into a single data matrix. miRNAs with a total sum of counts less than 100 copies across all samples were removed before further analyses. Differentially expressed miRNAs were identified using edgeR.^49^ Differentially expressed candidate miRNAs with a False Discovery Rate lower than 0.01 were retained.

### Biomarker validation phase

#### miRNA quantification by RT-qPCR

Complementary DNA (cDNA) was synthesised using miRCURY LNA RT Kit (Qiagen, MD, USA) following the manufacturer’s protocol and as previously reported.^50^ Custom miRCURY LNA miRNA PCR Assay (Qiagen, MD, USA) was used for RT-qPCR amplification of the selected miRNAs. The use of universal RT makes it possible to use one first-strand cDNA synthesis reaction as the template for multiple miRNA real-time PCR assays. In addition, both the forward and reverse PCR amplification primers are miRNA specific and optimisation with LNA provides exceptional sensitivity, extremely low background, and highly specific assays for better discrimination. miRCURY LNA SYBR® Green PCR Kit was used as a master mix for RT-qPCR amplification. The procedure was followed according to the manufacturer’s protocol and as previously reported,^50^ all samples were tested in duplicate and standard deviations of more than 1.000 between reactions were repeated. Uni sp6 spike in (Qiagen) was used across all samples as an internal quality control for evaluating the efficiency of cDNA synthesis and as an inter-plate calibrator in RT-qPCR. We employed three reference miRNAs to regulate variations, normalise small RNA input, and maintain quality control. Saliva samples with miRNA concentrations below 8 ng/µL and Ct values of reference genes exceeding 35 were excluded from the study.

#### miRNA normalisation strategy

The accurate quantification of miRNA expression levels and comparison between cohorts depends on appropriate normalisation to an endogenous miRNA. To date, there are no established endogenous miRNAs for normalisation of miRNA in saliva samples. Therefore, we selected five potential candidates namely, miR-16-5p, miR-191-5p, miR-484, SNORD 96 A and U6 snRNA from published literature.^16–18^ The best reference gene or combination of reference genes was evaluated using NormFinder software.^51^ We found that the commonly used U6 snRNA is unstable across tissue samples, thus we verified the stability of the above reference genes in tissue samples for normalising purposes.

#### miRNA in situ hybridisation

Unstained and hematoxylin and eosin (H&E) stained formalin-fixed paraffin-embedded (FFPE) tissue slides were obtained from the RBWH, and miRNA in situ hybridisation was performed using IsHyb In Situ Hybridisation (ISH) Kit following the manufacturer’s instructions (Biochain, San Francisco, California) and as previously reported.^52^ Proteinase K was purchased from Qiagen, MD, USA. hsa-miR-7-5p miRCURY LNA miRNA Detection probe (double DIG (Digoxigenin) labelled) (Qiagen) or negative control miRNA scrambled probe (double DIG labelled) (Integrated DNA Technologies) or u6 snRNA positive control probe (double DIG labelled) were used at 100 nmol/L per slide. Finally, slides were mounted with ProLong™ Gold Antifade Mountant (Thermo Fisher Scientific). Slides were scanned using Olympus BX63.

#### RT-qPCR data analysis

Normalised qRT-PCR data were obtained by calculating the difference between cycle threshold (Ct) values of the arithmetic mean of selected reference genes and target miRNAs (∆Ct). Statistical analyses were performed using JMP Pro (Version 17.0.0) and graphs were drawn using Graphpad Prism (Version 8). Biomarker panels were developed using LASSO logistic penalised regression with AICc (Akaike Information Criterion, corrected for small sample size) validation to balance model fit and number of parameters. Note that this method can include parameters with non-significant effect tests as long as their inclusion reduces the model’s AICc.

### Supplementary information

Supplementary Table 1

Supplementary Table 2

Supplementary Figure 1

## Data Availability

All data derived from public databases are available from the following site, Genomic Data Commons Data Portal (TCGA Dataset): https://portal.gdc.cancer.gov. All other data are available upon reasonable request from the corresponding author.
