# Supplementary figures and images for: A novel saliva-based miRNA profile to diagnose and predict oral cancer
Source: Int J Oral Sci. 2024 Feb 18;16:14. doi: 10.1038/s41368-023-00273-w (PMC10874410; doi:10.1038/s41368-023-00273-w)

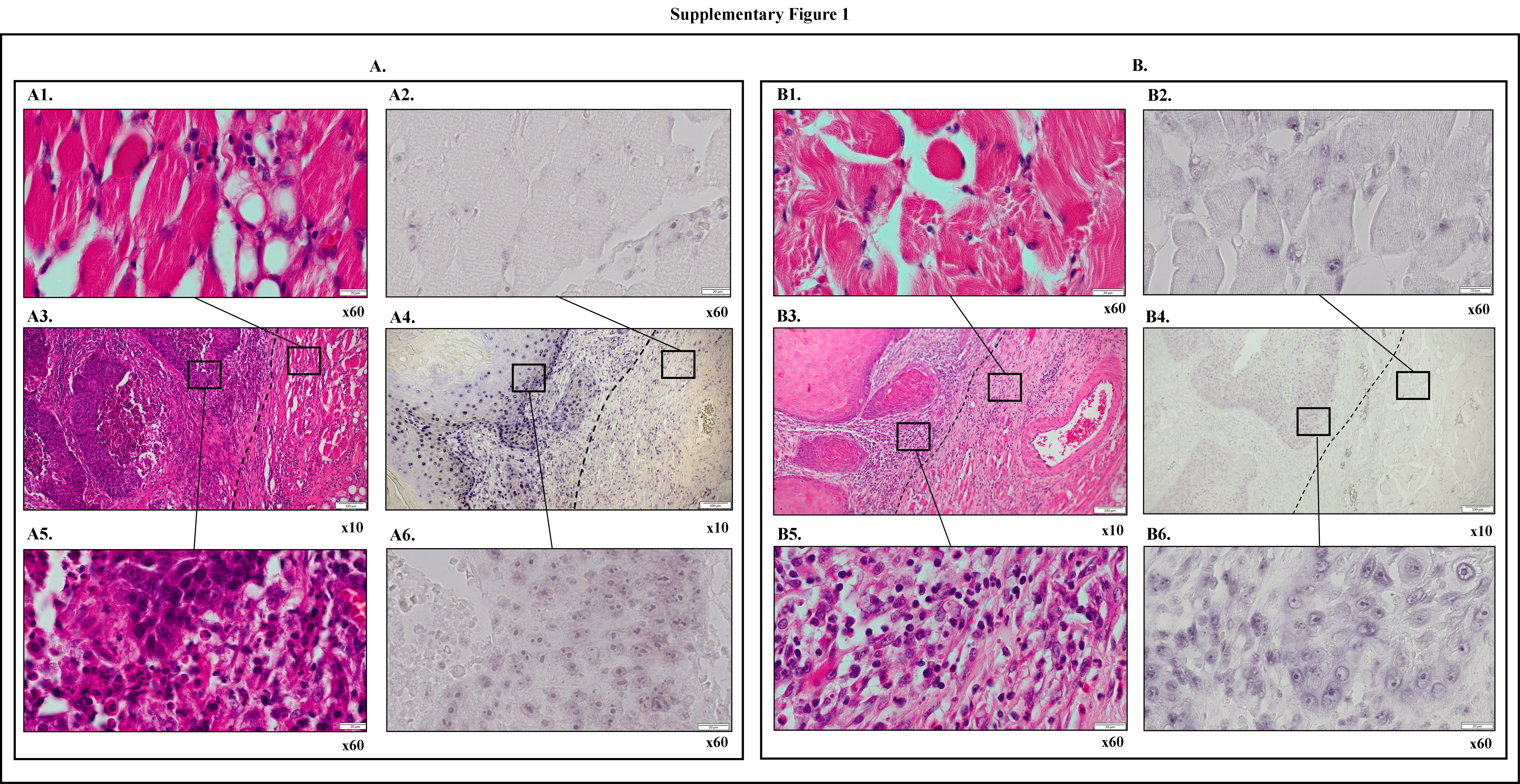

Supplement: Supplementary file 3 — Supplementary Figure 1 [file 41368_2023_273_MOESM3_ESM.tif]
